# A Novel Magnetically Levitated Intrapericardial Left Ventricular Assist System in Advanced Heart Failure—2-Year Results from a Prospective, Multi-Centre Study

**DOI:** 10.1093/ejcts/ezag014

**Published:** 2026-01-09

**Authors:** Xianqiang Wang, Haibo Chen, Juan Du, Xingtong Zhou, Liang Zou, Jinsong Huang, Liangwan Chen, Zhaoyun Cheng, Nianguo Dong, Xin Chen, Jincheng Liu, Yining Yang, Chunsheng Wang, Yifeng Yang, Zhenya Shen, Huishan Wang, Shengshou Hu

**Affiliations:** Department of Cardiovascular Surgery, National Center for Cardiovascular Diseases China: Chinese Academy of Medical Sciences & Peking, Union Medical College Fuwai Hospital, Beijing, 100037, China; Department of Cardiovascular Surgery, National Center for Cardiovascular Diseases China: Chinese Academy of Medical Sciences & Peking, Union Medical College Fuwai Hospital, Beijing, 100037, China; Department of Cardiovascular Surgery, National Center for Cardiovascular Diseases China: Chinese Academy of Medical Sciences & Peking, Union Medical College Fuwai Hospital, Beijing, 100037, China; Department of Cardiovascular Surgery, National Center for Cardiovascular Diseases China: Chinese Academy of Medical Sciences & Peking, Union Medical College Fuwai Hospital, Beijing, 100037, China; Department of Cardiovascular Surgery, National Center for Cardiovascular Diseases China: Chinese Academy of Medical Sciences & Peking, Union Medical College Fuwai Hospital, Beijing, 100037, China; Department of Cardiovascular Surgery, Guangdong Provincial People’s Hospital Guangdong Institute of Cardiovascular Diseases, Guangzhou, Guangdong, 510080, China; Department of Cardiovascular Surgery, Fujian Medical University Union Hospital, Fuzhou, Fujian, 350000, China; Department of Cardiovascular Surgery, Fuwai Central China Cardiovascular Hospital: Central China Fuwai Hospital of Zhengzhou University, Zhengzhou, Henan, 450003, China; Department of Cardiovascular Surgery, Huazhong University of Science and Technology Tongji Medical College, First Clinical College Union Hospital, Wuhan, Hubei, 430022, China; Department of Cardiovascular Surgery, Nanjing First Hospital, Nanjing, Jiangsu, 210006, China; Department of Cardiovascular Surgery, Fourth Military Medical University: Air Force Medical University, Xi’an, Shaanxi, 710032, China; Department of Cardiology, Xinjiang Medical University Affiliated First Hospital, Urumqi, Xinjiang, 830011, China; Department of Cardiovascular Surgery, Zhongshan Hospital Fudan University, Shanghai, 200032, China; Department of Cardiovascular Surgery, The Second Xiangya Hospital of Central South University, Changsha, Hunan, 410011, China; Department of Cardiovascular Surgery, First Affiliated Hospital of Soochow University, Soochow University, Suzhou, Jiangsu, 215006, China; Department of Cardiovascular Surgery, General Hospital of Northern Theatre Command, Shenyang, Liaoning, 110016, China; Department of Cardiovascular Surgery, National Center for Cardiovascular Diseases China: Chinese Academy of Medical Sciences & Peking, Union Medical College Fuwai Hospital, Beijing, 100037, China

**Keywords:** heart failure, mechanical circulatory support, left ventricular assist device, Corheart 6, outcomes

## Abstract

**Objectives:**

Left Ventricular Assist Devices have become an important therapy for advanced heart failure. We present 2-year results of the prospective, multicentre clinical trial in China, showing long-term efficacy and safety of a novel miniaturized magnetically levitated, continuous-flow left ventricular assist system.

**Methods:**

Patients were adults with a left ventricular ejection fraction <30% and a cardiac index <2.0 L/min/m^2^ without inotropic or dependence on continuous intravenous inotropes. Clinical data, including survival status, laboratory parameters, adverse events, and functional status, were collected at baseline and at 3, 6, 12, and 24 months and analysed. The primary end-point was the composite of survival at 2 years (on device support, transplant, or recovery), free of disabling stroke or device replacement.

**Results:**

A total of 50 patients were enrolled in 12 centres between January 2022 and July 2022. At the 2-year follow-up, 39 patients (78%) remained on device support, 3 patients (6%) received heart transplants, and 1 patient (2%) had the device explanted due to cardiac recovery. The 2-year event-free survival was 86% (95% confidence interval, 73.3-94.2). Major adverse events included right heart failure (*n* = 2, 4%), stroke (*n* = 3, 6%), driveline infection (*n* = 6, 12%), and gastrointestinal bleeding (*n* = 2, 4%). No haemolysis or device malfunction occurred.

**Conclusions:**

This study demonstrates promising long-term effectiveness and safety of the Corheart 6 left ventricular assist system for circulatory support in patients with advanced heart failure.

**ClinicalTrials.gov:**

NCT05353816

## Graphical abstract

**Figure:**
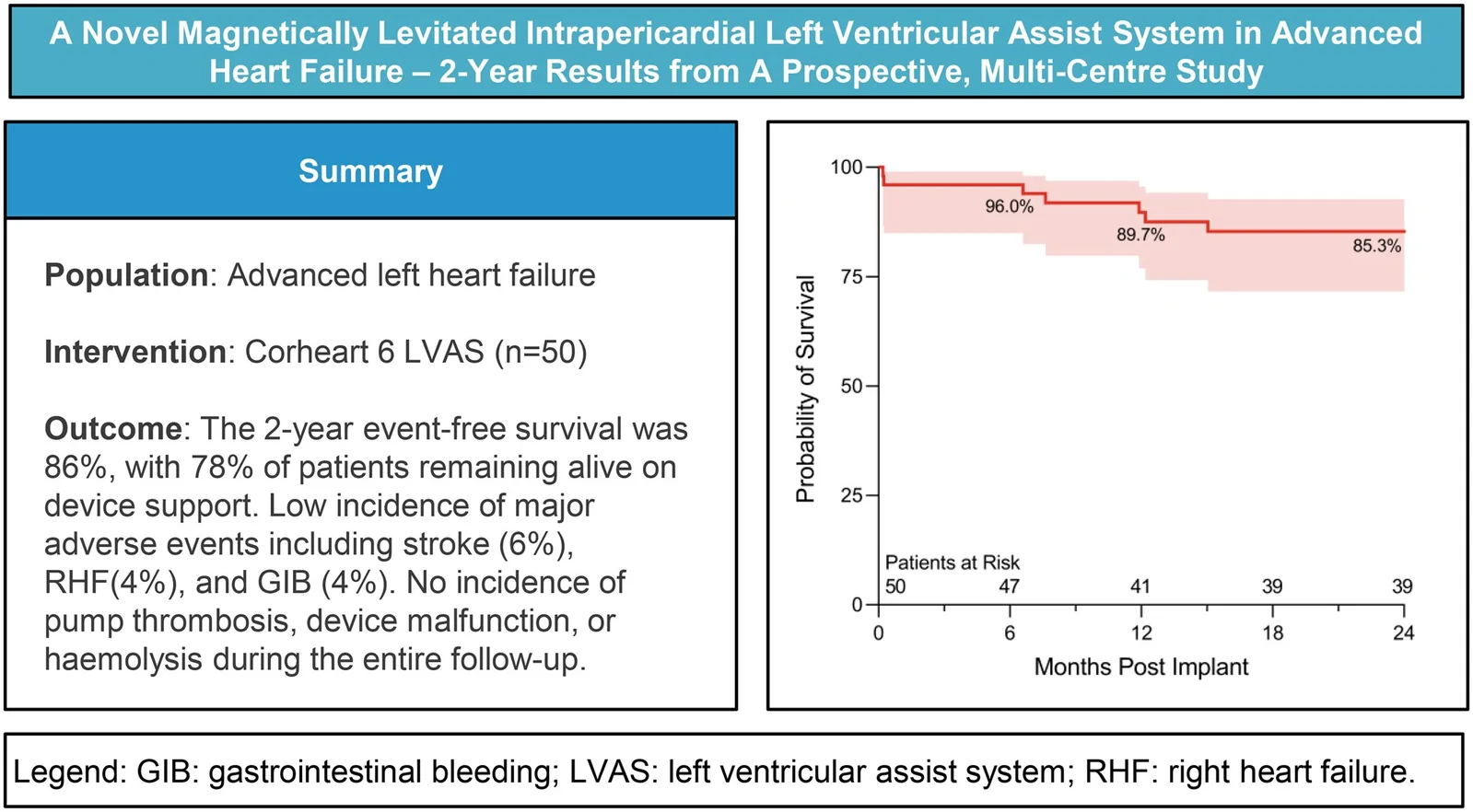

## Introduction

Heart failure (HF) is a major global health condition, affecting over 56 million people worldwide and imposing a significant public health burden.^1^

Despite significant advancements in HF pharmacotherapy, a proportion of patients refractory to optimal guideline-directed medical therapy (GDMT) progress into advanced HF.^2–4^ This terminal stage of HF carries a poor prognosis. Heart transplantation is considered the gold standard treatment, but the scarcity of donor hearts severely limits its widespread applicability. Consequently, durable left ventricular assist devices (LVADs) are widely utilized in clinical practice.

Advancements in LVAD technology, particularly with the introduction of magnetically levitated pumps such as the HeartMate 3 (HM3, Abbott), have led to improved outcomes for recipients, including survival rates and quality of life.^5–7^ However, a gap persists between current-generation LVADs and the ideal LVAD, with areas for improvement such as miniaturization and hemocompatibility.^8^

The Corheart 6 left ventricular assist system (LVAS; Shenzhen Core Medical Technology Co., Ltd) is a next-generation LVAD. Compared with current-generation LVADs, the Corheart 6 incorporates fully magnetically levitated technology and concurrently features a significantly minimized pump size, with a diameter of 34 mm, a width of 26 mm, and a weight of 90 g.

The pivotal trial was designed to evaluate the 3-month effectiveness and safety and enrolled 50 patients between January 2022 and July 2022. The trial results led to approval by the Chinese National Medical Products Administration (NMPA) in 2023. Following completion of the pivotal trial’s primary end-point assessment, patient follow-up was extended to 2 years. This study aimed to analyse the data collected through 2 years of follow-up to evaluate the long-term effectiveness and safety.

## Methods

### Study designed

This prospective, multicentre, single-arm study was conducted at 12 centres located in China, with Fuwai Hospital serving as the principal coordinating centre. The study conforms with the Declaration of Helsinki, the International Conference on Harmonization (ICH) Good Clinical Practice (GCP), Regulations of the People’s Republic of China on Clinical Trials of Medical Devices (Bureau Order No. 5), and country-specific requirements in China. All participating centres obtained approval from their respective ethical committees. This study was registered on ClinicalTrials.gov (NCT05353816).

Major inclusion criteria included: age ≥18 years, body surface area (BSA) ≥1.0 m^2^, and advanced left heart failure. Key exclusion criteria included: irreversible end-organ dysfunction, active infection, planned biventricular support, pregnancy, or intolerance to anticoagulation or antiplatelet therapies. Detailed inclusion criteria, exclusion criteria, and other clinical trial design information are provided in the Supplemental Materials. All patients provided written informed consent. Following the enrolment of all patients, the follow-up duration was extended to 2 years post-implantation; this modification received approval from the institutional ethics committee of Fuwai Hospital.

The primary endpoint was defined as a composite of survival to transplant, recovery, or LVAS support free of disabling stroke (modified Rankin score > 3) or reoperation to replace the pump due to pump malfunction. The sample size was calculated based on an expected 3-month survival of 80% versus a minimum acceptable rate of 60%. Considering an anticipated dropout rate of approximately 10%, the final sample size was set at least 50 patients. Secondary end-points included the functional status as assessed with the New York Heart Association (NYHA) classification and the 6-minute walk test (6MWT). Quality of life was assessed with the EuroQol 5-dimension 3-level questionnaire (EQ-5D-3L), the EQ-5D Visual Analogue Scale (EQ-5D VAS), and the Kansas City Cardiomyopathy Questionnaire (KCCQ). Patients’ data were collected at baseline and at 3, 6, 12, and 24 months post-implantation. Adverse events (AEs) definitions as described by the Interagency Registry for Mechanically Assisted Circulatory Support (INTERMACS) guidelines were employed.^9^ Patients were followed to the primary end-point at 2 years or other outcomes (death, explant or transplant), which occurred first. The 24-month follow-up for all patients was completed by September 2024.

### Device description

The Corheart 6 is a next generation, miniature magnetically levitated centrifugal pump developed by Shenzhen Core Medical Technology Co., Ltd. The pump features an integrated magnetic levitation design that contributes to its compact dimensions: a diameter of 34 mm, a thickness of 26 mm, and a weight of only 90 g (**Figure 1A** and **B**). This miniaturization allows for a broad clinical treatment range, accommodating patients with a BSA as low as 1.0 m^2^, and potentially facilitates minimally invasive surgical approaches, smaller incisions, and rapid patient recovery. Leveraging its novel miniaturized magnetically levitated technology, the Corheart 6 is designed for enhanced hemocompatibility and effective blood washout. This is achieved by minimizing blood exposure to non-physiological shear stress and artificial biomaterial surfaces. The secondary flow pathways incorporate a short, ‘straight-line’ configuration, which can enhance washout, shorten blood residence time, and minimal blood damage within the secondary flow area. This advanced flow channel design, along with the low shear stress distribution, mitigates blood damage induced by non-physiological shear stress and reduces the risk of thrombus formation (**Figure 1C**). The pump’s operational speed control ranges from 2200 to 4300 RPM, delivering a total system flow up to 10.0 L/min.

**Figure 1.**
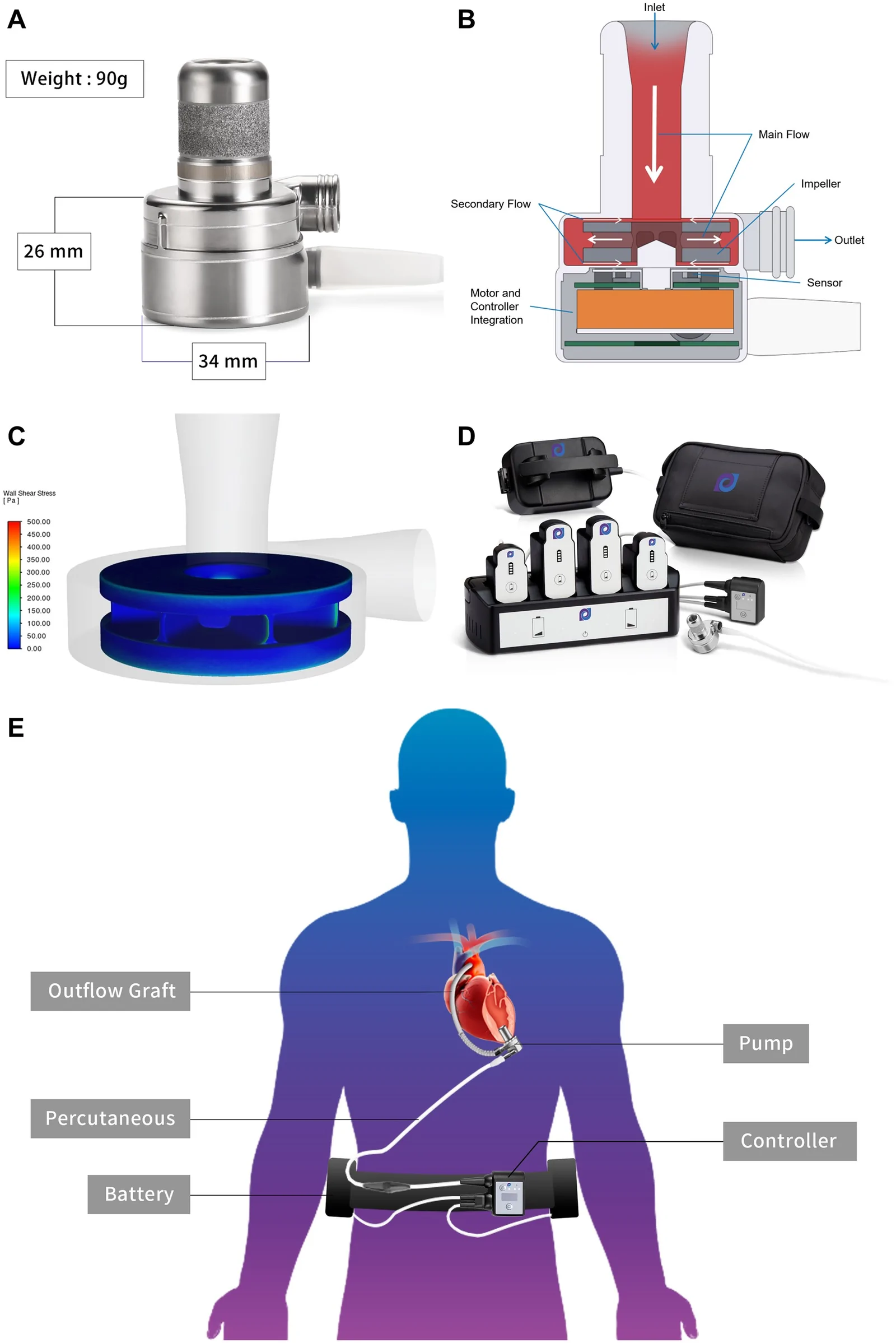
(A) The Corheart 6 Blood Pump, (B) Magnetic Bearing and Electric Motor Structure, (C) Shear Force Distribution of Corheart 6 pump based on fluid simulation results, (D) The Components of Corheart 6, Including Pump, Controller, Rechargeable Lithium Battery Pack, Charging Stand, Power Adapter, and Portable Bag, (E) An Overall Schematic of the Corheart 6 Implantation. The Pump is Implanted at the Apex of the Heart and Connected to an External Controller via a Percutaneous Cable. The external Controller is Powered by 2 Lithium Batteries, Which Provide Energy to the Entire System.

The Corheart 6 implantation was accomplished via median sternotomy under cardiopulmonary bypass. The pump was positioned within the pericardial space, with the inflow cannula secured to the left ventricular apex and the outflow graft anastomosed to the ascending aorta. The outlet graft sleeve (**Figure S3**), placed around the outflow graft, features a hollowed design to prevent compression or bending of the outflow graft by surrounding tissue. A percutaneous cable connects the pump to the external controller, which has 2 power supply modes: 2 power cables linked to 2 sets of rechargeable lithium batteries, or 1 set of batteries and a power adapter (**Figure 1D** and **E**). The device incorporates an advanced disc motor design, which is engineered to reduce power consumption and prolong battery life compared to systems with similar capacities, thereby potentially minimizing restrictions on patient mobility.

The anticoagulation protocol was developed based on international guidelines,^10^ the LVAD management experience at Fuwai Hospital, and findings from pre-clinical animal studies.^11^ Anticoagulation was started with intravenous heparin, usually at 12-24 hours post-operation, when the chest tube drainage was less than 40 mL/hours over a 2-3 hour period, and was titrated to an aPTT of 45 to 60 seconds. When patients were stable and showed no signs of active bleeding, warfarin and aspirin (100 mg daily) were started, usually at 2-3 days post-operation. Intravenous heparin was discontinued upon achieving a target international normalized ratio (INR) of 2.0 to 2.5 with warfarin.

### Statistical analysis

Descriptive analyses were performed for all variables. Continuous variables were summarized as mean and standard deviation (SD), or median (interquartile range, IQR), according to normality assessed by the Shapiro-Wilk test. Non-normally distributed variables are reported as median (IQR). Categorical variables were presented as counts and percentages. Overall survival was analysed using Kaplan-Meier methods, censoring at heart transplantation or device explantation for recovery. Multivariable competing-risk analyses were performed as sensitivity analysis, treating death, transplantation and explantation for recovery as competing events. Adverse events were summarized as the number and percentage of patients within the early (0 to 90 days), late (91 to 730 days), and overall 2-year follow-up. Events other than all-cause death were analysed using cumulative incidence functions estimated by Aalen-Johansen method. Postoperative parameters were compared to the baseline using the paired Student’s *t*-test or Wilcoxon Signed Rank Test. Changes in quality of life measures, including EQ-5D-3L, EQ-5D VAS, and KCCQ score, from the baseline were analysed using mixed effects linear regression models, with the change from the baseline as the dependent variable and baseline score and visit as covariates. A 2-sided *P *< 0.05 was considered statistically significant. All analyses were performed using SPSS Statistics version 25.

## Results

### Baseline characteristics

A total of 50 patients were enrolled in this pivotal study. All patients were diagnosed with advanced left heart failure and were classified as NYHA Class IV. The indications for implantation were Bridge-to-Transplantation (BTT) in 16 (32%) patients and Destination Therapy (DT) in 34 (68%) patients. Detailed demographics and baseline characteristics are provided in **Table 1**.

**Table 1.** Baseline Characteristic

Characteristic	*N* = 50
Age [years, mean (SD)]	50.4 (10.1)
Male [n (%)]	41 (82%)
BSA [m^2^, mean (SD)]	1.9 (0.2)
Pre-operative risk factors [n (%)]	
Atrial fibrillation	11 (22%)
Ventricular fibrillation/tachycardia	14 (28%)
Hypertension	9 (18%)
Stroke	4 (8%)
Valve insufficiency/regurgitation	35 (70%)
Myocardial infarction	3 (6%)
Primary heart failure aetiology [n (%)]	
Dilated cardiomyopathy	38 (76%)
Ischaemic aetiology	10 (20%)
Other	2 (4%)
LVEF [%, mean (SD)]	23.9 (3.8)
LVEDD [mm, mean (SD)]	77.7 (9.3)
TAPSE [mm, mean (SD)]	15.3 (2.9)
CI [L/min/m^2^, median (IQR)]	1.7 (1.2, 2.1)
PCWP [mmHg, median (IQR)]	15 (13, 20)
CVP [mmHg, mean (SD)]	7.9 (4.8)
Arterial blood pressure [mmHg, mean (SD)]	
Systolic	100.5 (13.7)
Diastolic	66.5 (9.9)
Pulmonary artery pressure [mmHg, median (IQR)]	
Systolic	33.5 (27.0, 46.0)
Diastolic	17.0 (13.0, 26.0)
Mean	24.0 (19.0, 32.0)
NYHA Class IV [n (%)]	50 (100%)
Pre-operative MCS support [n (%)]	
IABP	3 (6%)
ECMO	1 (2%)
Administration of inotropic and/or vasoactive agents [n (%)]	50 (100%)

Abbreviations: BSA, body surface area; CI, cardiac index; CVP, central venous pressure; ECMO, extracorporeal membrane oxygenation; IABP, intra-aortic balloon pump; IQR, interquartile range; LVEDD, left ventricular end diastolic diameter; LVEF, left ventricular ejection fraction; MCS, mechanical circulation support; NYHA, New York Heart Association; PCWP, pulmonary capillary wedge pressure; SD, standard deviation; TAPSE, tricuspid annular plane systolic excursion.

### Outcomes

All 50 patients successfully underwent implantation of the Corheart 6 via median sternotomy under cardiopulmonary bypass. Surgical procedures were uneventful. 18 (36%) patients received isolated Corheart 6 implantation, while 25 (50%) patients underwent concomitant valvular or coronary artery surgeries. The mean cross-clamp time was 70.58 ± 36.37 min, and the mean cardiopulmonary bypass time was 138.67 ± 73.59 min. At intensive care unit (ICU) admission, the mean pump speed setting was 2660 ± 379 RPM, achieving a mean flow rate of 3.6 ± 1.7 L/min. The further details are shown in **Table S1**.

Two-year follow-up was completed for all patients, with no loss to follow-up. At 3 months post-implantation, the event-free survival was 96.0%, with the 95% CI ranging from 86.3% to 99.5%. At 2 years after implantation, the event-free survival was 86.0%, with the 95% CI ranging from 73.3% to 94.2%, which is a composite of 39 (78%) patients remaining on original device support, 3 (6%) patients undergoing heart transplant, and 1 (2%) patient with device explantation due to cardiac recovery.Among the transplanted patients, 2 patients received transplantation for recurrent ventricular fibrillation, and 1 patient was transplanted as intended for BTT after waiting for a suitable donor heart. A total of 7 patients (14%) died during the 2-year period. Two deaths (4%) occurred in the perioperative period (one due to severe arrhythmia, one due to right heart failure). Five deaths (10%) occurred during long-term follow-up. One was due to right heart failure, 2 were attributed to haemorrhagic stroke, and 2 resulted from multi-organ failure. All mortalities were not related to the implantation procedure or device malfunction. The competing outcomes are shown in **Figure 2A** and the Kaplan–Meier survival curve is shown in **Figure 2B**. The median duration of device support at 2-year follow-up was 726 days.

**Figure 2.**
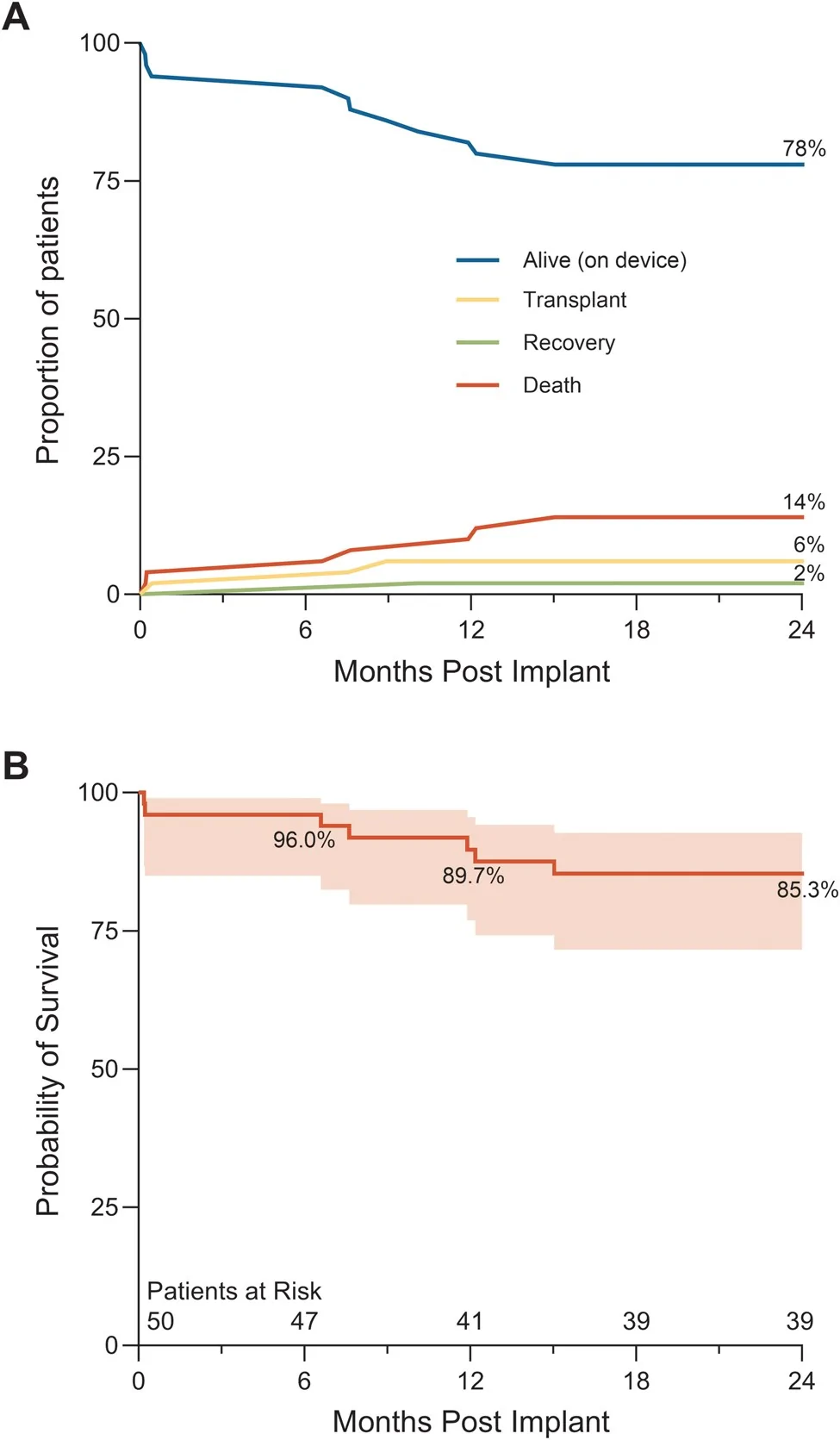
(A) The Competing Risk Plot for Death, Transplant, Recovery, and Ongoing Support During the 24 Months Follow-Up, (B) 24 Months Kaplan–Meier Survival Analysis After Corheart 6 Implantation. Abbreviation: CI, confidence interval.

At the 2-year follow-up visit, the mean pump flow was 3.4 ± 0.8 L/min, and the mean pump speed was 2881 ± 209 RPM. Laboratory tests reflecting organ function were presented in **Table S2**. Lactate dehydrogenase (LDH) level slightly declined after 1 month and remained stable at a normal level from 3 months to 24 months (**Figure S6**). INR values were maintained within a low-level therapeutic range throughout the Corheart 6 support period (**Figure S7**).

Significant improvements in quality of life were observed across all assessed methods. Scores on the KCCQ, EQ-5D-3L, and EQ-5D VAS all improved significantly from baseline to 3 months post-implantation (all *P *< 0.01), and these improvements were sustained through 24 months post-implantation (**Figure 3A-C**). Physical functioning was assessed by the NYHA classification and 6MWT. All patients were NYHA Class IV at baseline. By 6 months post-implantation, a significant majority had improved to NYHA Class I or II (**Figure 4A**). The 6MWT distance significantly improved from baseline at 3 months (*P *< .01, **Figure 4B**), with evidence of further improvement at 12 months.

**Figure 3.**
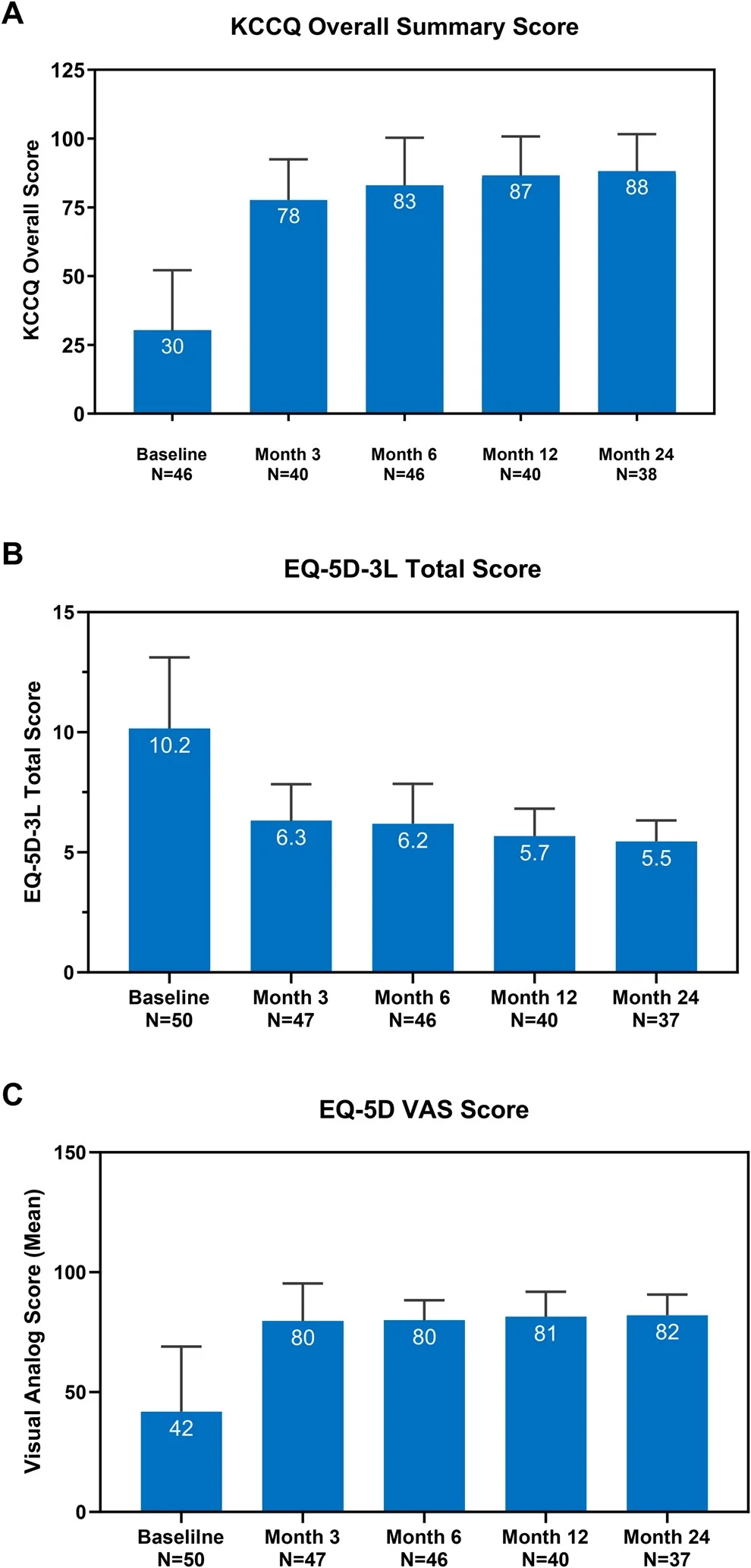
Quality of Life Outcomes from Baseline to 24 Months Post-Implantation. (A) Kansas City Cardiomyopathy Questionnaire (KCCQ) Overall Summary Scores, (B) EuroQol 5-dimension 3-level Questionnaire (EQ-5D-3L) Total Scores, (C) EQ-5D Visual Analogue Scale (VAS) Scores.

**Figure 4.**
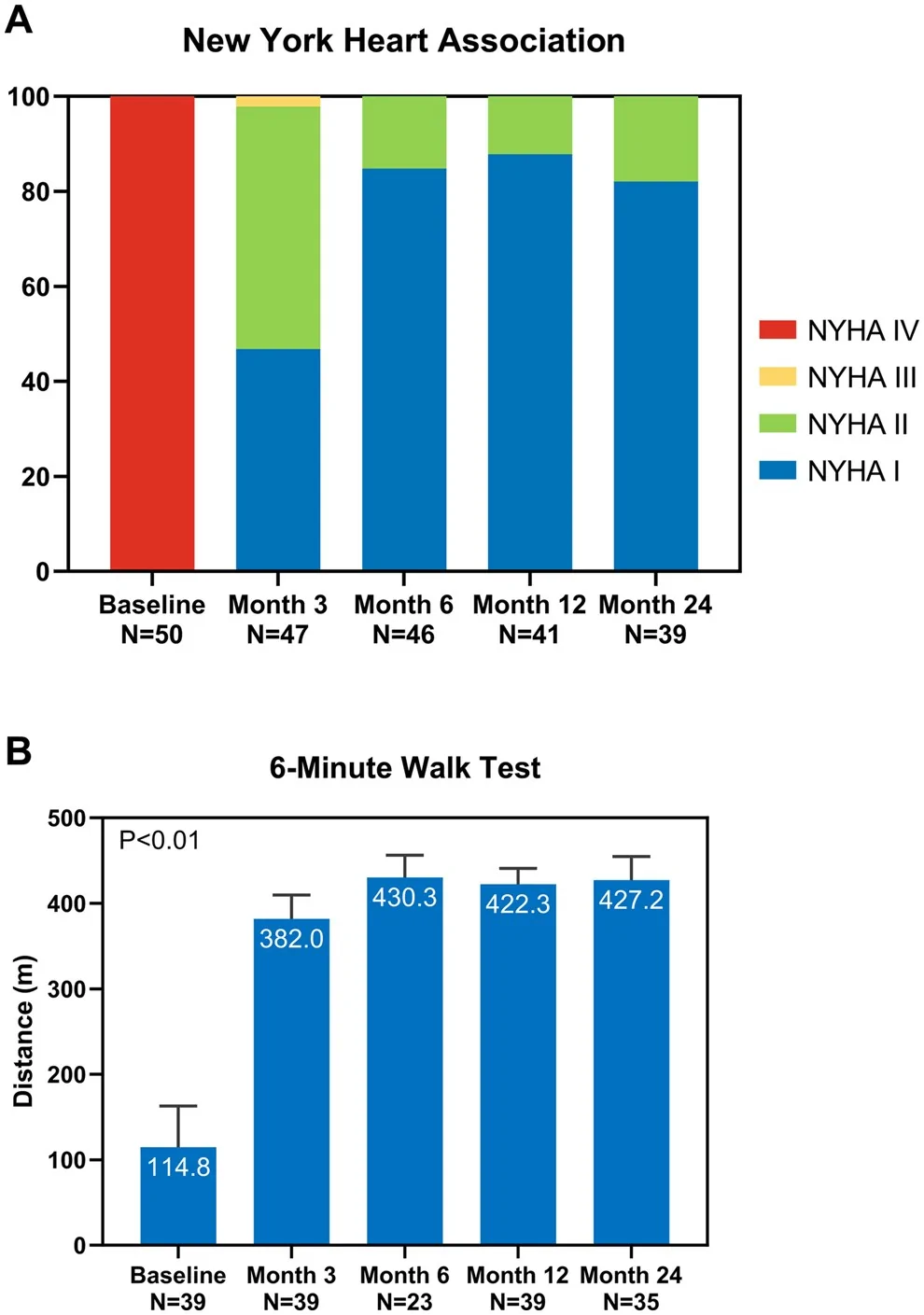
(A) Percentage of Patients in New York Heart Association (NYHA) Classification at Each Follow-Up Visit, (B) Six-Minute Walk Test (6MWT) Results at Each Follow-Up Visit. Patients Who Were Unable to Complete the 6MWT Due to Heart Failure were Assigned a Walking Distance of 0 m for Analysis.

### Adverse events

The rates of AEs observed in this study are reported in **Table 2**. Notably, there were no reported incidences of haemolysis, device malfunction, outflow graft obstruction, or pump thrombosis. Cardiac arrhythmia and infection were the most common adverse events. Overall, cardiac arrhythmia occurred in 17 patients (34%). The incidence was distributed between the early and late periods, affecting 12 patients (24%) and 8 patients (16%), respectively. All arrhythmic events were successfully managed with medical therapy or electrical cardioversion. Stroke occurred in 3 patients (6%) was haemorrhagic. Other major AEs included driveline infection in 6 patients (12%), sepsis in 1 patient (2%), right heart failure (RHF) in 2 patients (4%), and gastrointestinal bleeding in 2 patients (4%).

**Table 2.** Summary of Adverse Events

Adverse events^a^ (*N* = 50)	Time period			Events per 100 patient-years
	≤90 days	>90 days	Total	
Infection				
Sepsis	0% (0,0)	2% (1,1)	2% (1,1)	1.15
Driveline infection	0% (0,0)	12% (6,9)	12% (6,9)	10.39
Major bleeding				
Bleeding required surgery	2% (1,1)	0% (0,0)	2% (1,1)	1.15
Bleeding required transfusion	2% (1,1)	2% (1,1)	4% (2,2)	2.31
Gastrointestinal bleeding	0% (0,0)	4% (2,2)	4% (2,2)	2.31
Neurologic dysfunction				
Haemorrhagic stroke	0% (0,0)	6% (3,3)	6% (3,3)	3.46
Ischaemic stroke	0% (0,0)	0% (0,0)	0% (0,0)	0.00
Transient ischaemic attack	0% (0,0)	0% (0,0)	0% (0,0)	0.00
Cardiac arrhythmia	24% (12,19)	16% (8,11)	34% (17,30)	34.64
Right heart failure	2% (1,1)	2% (1,1)	4% (2,2)	2.31
Renal dysfunction	12% (6,6)	2% (1,1)	14% (7,7)	8.08
Respiratory failure	0% (0,0)	2% (1,1)	2% (1,1)	1.15
Hepatic dysfunction	8% (4,4)	0% (0,0)	8% (4,4)	4.62
Psychiatric episodes	2% (1,1)	0% (0,0)	2% (1,1)	1.15
Venous thromboembolism	6% (3,3)	0% (0,0)	6% (3,3)	3.46
Haemolysis	0% (0,0)	0% (0,0)	0% (0,0)	0.00
Pump thrombosis	0% (0,0)	0% (0,0)	0% (0,0)	0.00
Device malfunction	0% (0,0)	0% (0,0)	0% (0,0)	0.00

a % patients (# patients, # events).

## Discussion

This study represents good long-term performance and safety of the Corheart 6 in patients with advanced HF in China. The overall 2-year composite survival rate was comparable to the reported survival rates of other LVAD studies, which typically range from 74.0% to 79.5% at similar follow-up durations.^12–14^ A significant improvement in quality of life was observed among all patients as also in the MOMENTUM 3 trial.^13^ The lightweight and small extracorporeal components and the extended battery life of the system, which can last up to 33 hours, contribute to greater patient mobility and reduced lifestyle restrictions.

AEs incidence is a critical assessment of LVAD safety, particularly hemocompatibility-related and device-related complications such as haemolysis, bleeding, pump thrombosis, and driveline infection.^15^^,^^16^ In this study, the Corheart 6 demonstrated a favourable hemocompatibility profile over the 2-year follow-up, with no instances of clinically significant haemolysis or pump thrombosis observed. This finding was further supported by LDH levels, an indicator of haemolysis, which remained within a normal range throughout the study. This enhanced hemocompatibility may be attributed to the blood pump’s internal fluid path design, which reduces the surface area of blood contact, shear stress within the pump, and blood residence time, thereby minimizing blood “damage” and improving blood compatibility.^17^ Furthermore, a particularly noteworthy aspect of this study was the absence of any thrombotic events throughout the 2-year follow-up, despite the maintenance of a lower intensity anticoagulation regimen, with a median INR of 1.96 (IQR: 1.69-2.33). While previous studies have demonstrated the feasibility of conservative anticoagulation strategies in the early postoperative period for the HeartMate 3,^18^^,^^19^ our study demonstrated that the Corheart 6, which exhibits enhanced hemocompatibility, maintaining a lower anticoagulation intensity is safe over the long term in the Chinese population.

Neurologic dysfunction (including ischaemic stroke and haemorrhagic stroke) is an important hemocompatibility-related adverse event. In this study, 3 patients (6%) experienced haemorrhagic strokes, all occurring more than 90 days, and no ischaemic strokes were observed. The contributing factors for these haemorrhagic events included infection (*n* = 2) and over-anticoagulation (*n* = 1). The infection-related strokes were not associated with driveline infection, one was due to COVID-19 and one was due to the sepsis caused by recurrent lung infections. It is worth noting that there did not occur ischaemic stroke although low anticoagulation.

Infection is common after LVAD surgery and is associated with an increased risk of stroke and mortality.^20^^,^^21^ In this study, 2 haemorrhagic strokes were attributed to preceding infections, consistent with findings from previous studies. Regarding specific infection types, driveline infections were identified in 6 patients (12%) beyond 90 days post-implantation. All patients achieved favourable outcomes with enhanced treatment protocols. Sepsis, in contrast, was observed in only 1 patient (2%) and was secondary to recurrent pulmonary infection.

Bleeding is also a common complication following LVAD implantation, particularly gastrointestinal bleeding.^22^ In the first prospective cohort study of HM3 and the MOMENTUM 3 trial, the reported incidence of any bleeding post-HM3 implantation was 50.0% and 43.7%, respectively, while gastrointestinal bleeding was 20.0% and 24.5%, respectively.^12^^,^^23^ In contrast, our study demonstrated a lower overall incidence of bleeding of 10% and gastrointestinal bleeding of 4%. These rates are numerically lower than those reported for the HM3. Furthermore, the incidence of bleeding requiring surgical intervention in our study (2%) also appeared lower than the rates reported for the HM3 (9.7% to 16.0%). This potentially favourable bleeding profile with the Corheart 6 may be attributable to its compact pump size, which could minimize trauma to surrounding tissues during implantation.

The incidence of RHF was low, with only 2 patients (4%) experiencing this complication. One case occurred in the perioperative period and was attributed to limited experience with patient selection at the early stage of the trial. The other case developed prior to the 12-month follow-up visit and was likely related to insufficient patient self-management of volume during the prolonged interval between scheduled visits. Following these 2 events, we enhanced training for preoperative risk assessment and intensified postoperative education on volume management. It is plausible that these interventions contributed to the subsequently low overall incidence of RHF observed in this study.

### Current status and future development

As of October 2025, over 1000 Corheart 6 devices have been implanted worldwide. This rapid clinical adoption is closely linked to the promising outcomes observed in clinical use, as demonstrated in this present study. While the device represents a significant advancement, persistent challenges like driveline infections and RHF remain.

The device’s unique characteristics—compact size, low weight, and favourable hemocompatibility—make it ideal for right-side implantation. This presents significant potential for its use as a right ventricular assist device, offering a novel therapy for managing RHF or biventricular failure. Notably, it has been successfully used in a biventricular configuration in China.^24^ Moreover, the integration of an intelligent control algorithm is a promising advancement for LVADs. By adjusting pump speed according to the patient’s real-time needs, such a system may reduce post-LVAD RHF risk and optimize support. The future addition of this capability to the Corheart 6 is a key development goal.

## Limitations

Several limitations of the present study should be acknowledged. Firstly, this study was non-randomized and non-blinded, without a control group, and thereby preventing head-to-head comparisons with contemporary devices. Secondly, several participating centres had limited prior clinical experience with LVAD implantation before initiating this investigation. Although mitigated through standardized operative training programs to reduce the potential heterogeneity, it is necessary to consider the learning curve when interpreting clinical end points due to inter-centre differences. Thirdly, this study with a sample size of 50 participants may be considered limited in scope.

## Conclusion

In conclusion, this study of the Corheart 6 in patients with advanced heart failure found a 2-year composite survival of 86%, with statistically significant improvements in functional capacity and quality of life and no reported instances of pump thrombosis. These findings support the Corheart 6 as a viable option for long-term mechanical circulatory support in patients with advanced heart failure.

## Supplementary Material

ezag014_Supplementary_Data

## Data Availability

The data of this study will be shared on reasonable request to the corresponding author.
