# Supplementary material for: A Novel Magnetically Levitated Intrapericardial Left Ventricular Assist System in Advanced Heart Failure—2-Year Results from a Prospective, Multi-Centre Study
Source: Eur J Cardiothorac Surg. 2026 Jan 9;68(11-12):ezag014. doi: 10.1093/ejcts/ezag014 (PMC13769434; doi:10.1093/ejcts/ezag014)
Supplement: Supplementary file 1 [file ezag014_supplementary_data.zip › Supplementary_Material.docx]

**Supplemental Materials**

[**Supplementary Materials & Methods**	2](#_Toc213873533)

[Definitions of inclusion and exclusion criteria	2](#_Toc213873534)

[**Supplementary Tables**	6](#_Toc213873535)

[Table S1 Operative Characteristic	6](#_Toc213873536)

[Table S2 Laboratory Values	7](#_Toc213873537)

[Table S3 Linear Mixed-Effected Regression Analyses of Quality of Life Change	10](#_Toc213873538)

[**Supplementary Figure**	12](#_Toc213873539)

[Figure S1 Corheart® 6 Head Pressure Flow Rate (HQ) Curves.	12](#_Toc213873540)

[Figure S2 Prospective, Multi-Centre Study Flow Diagram	13](#_Toc213873541)

[Figure S3 The Outlet Graft sleeve of Corheart 6 Left Ventricular Assist System	14](#_Toc213873542)

[Figure S4 Cumulative Incidence of Right Heart Failure	15](#_Toc213873543)

[Figure S5 Cumulative Incidence of Driveline Infection	16](#_Toc213873544)

[Figure S6 Dynamic changes in lactate dehydrogenase (LDH) levels from baseline to 24 months post-implantation.	17](#_Toc213873545)

[Figure S7 Distribution of International Normalized Ratio (INR) values at baseline and at different follow-up visit (1-month, 3-month, 6-month, 12-month and 24-month) post-implantation.	18](#_Toc213873546)

**Supplementary Materials & Methods**

**Definitions of inclusion and exclusion criteria**

**Inclusion Criteria:**

1. Age ≥ 18 years
2. Patient or legal representative has signed Informed Consent Form (ICF)
3. BSA ≥ 1.0 m^2^
4. Females of child bearing age must agree to use adequate contraception
5. Patients with NYHA Class IV heart failure symptoms who have failed to be reversed by previous standardized oral treatment with anti-heart failure drugs (ACEI, beta-blockers and diuretics)
6. LVEF ≤ 30%, and at least one of the following conditions occurs:
   a. For those whose condition cannot be reversed or can't be removed by using intra-aortic balloon pump (IABP), extracorporeal membrane oxygenator (ECMO) or other short-term mechanical circulation assistance;
   b. Rely on continuous intravenous administration of one or more cardioactive drugs or positive inotropic drugs;
   c. Meeting diagnostic criteria of cardiogenic shock: blood pressure < 90/60mmHg; Cardiac discharge index < 2.0; Pulmonary capillary wedge pressure > 18mmHg

**Exclusion Criteria:**

1. Etiology of HF due to or associated with uncorrected thyroid disease, obstructive cardiomyopathy pericardial disease, amyloidosis or restrictive cardiomyopathy.
2. Active, uncontrolled infection confirmed by a combination of clinical symptoms and laboratory tests.
3. In the investigator's judgment, there are technical difficulties that lead to an unacceptable hig surgical risk.
4. The patient was intolerant to anticoagulant or antiplatelet therapy or was unable to receive other perioperative and postoperative treatments that the investigator deemed necessary based on the patient's health condition.
5. Patients require biventricular assist device support.
6. Pregnancies.
7. The patient had moderate to severe aortic insufficiency or a history of mechanical aortic valve implantation, but did not agree or could not be corrected by replacement or replacement of biological valve during LVAD implantation.
8. History of visceral organ transplantation.
9. Have uncorrected thrombocytopenia or severe coagulopathy, such as diffuse intravascular coagulation.
10. TBIL (total bilirubin) > 3.0 mg/dL and serum creatinine (SCr) > 3.0 mg/dL within 48 hours before surgery may require dialysis.
11. History of severe chronic obstructive pulmonary disease (COPD) or restrictive lung disease or a diagnosis of primary pulmonary hypertension.
12. Pulmonary embolism and pulmonary artery systolic blood pressure exceeding 60mmHg within 3 weeks prior to enrollment combined with at least one of the following 2 parameters demonstrated that pulmonary vascular resistance did not respond to drug therapy: pulmonary vascular resistance greater than 8 wood units; The transpulmonary differential pressure exceeds 20mmHg.
13. Established and untreated abdominal or thoracic aortic aneurysm > 5cm in diameter.
14. Severe peripheral vascular disease with resting pain or extremity ulceration.
15. Patients with psychiatric disorders/disorders, irreversible cognitive impairment or psychosocial problems are at risk of failing to comply with research protocols and regulations governing the use of implantable LEFT ventricular assist systems or brain death from various causes.
16. Expect to live less than 1 year if you have a malignant tumor or other disease.
17. Patients participate in any other clinical trials that may influence the results of this study.
18. Other circumstances that are unforeseen and determined by the researcher to be unsuitable.

**Supplementary Tables**

**Table S1 Operative Characteristic**

| **Characteristic** | **N=50** |
| --- | --- |
| Isolated LVAD implantation [n (%)] | 18 (36%) |
| Concomitant Surgical Procedure | |
| Left CABG [n (%)] | 5 (10%) |
| Right CABG [n (%)] | 1 (2%) |
| Mitral valvuloplasty [n (%)] | 15 (30%) |
| Tricuspid valvuloplasty [n (%)] | 6 (12%) |
| Aortic valve replacement [n (%)] | 4 (8%) |
| Left atrial appendage exclusion [n (%)] | 9 (18%) |
| Patent foramen ovale closure [n (%)] | 2 (4%) |
| Cardiopulmonary bypass time [minutes, Median (IQR)] | 118.0 (93.0, 157.8) |
| Cross-clamp time [minutes, Mean (SD)] | 73.13 (38.80) |
| Postoperative ICU length of stay [days, Median (IQR)] | 10.5 (7.0, 15.8) |
| Postoperative hospital length of stay [days, Median (IQR)] | 29.0 (26.0, 37.0) |
| LVAD settings at ICU admission | |
| Flow rate [L/min, Mean (SD)] | 3.6 (1.7) |
| Pump speed [rpm, Mean (SD)] | 2660 (379) |

Abbreviations: CABG, coronary artery bypass grafting; LVAD, left ventricular assist device; ICU, intensive care unit.

**Table S2 Laboratory Values**

| **Variable** | **Visit** | **N** | **Mean (SD)** |
| --- | --- | --- | --- |
| **RBC (×10^12^/L)** | Baseline | 50 | 4.72 (0.68) |
|  | Month 3 | 47 | 4.62 (0.62) |
|  | Month 6 | 44 | 4.84 (0.62) |
|  | Month 12 | 39 | 4.91 (0.67) |
|  | Month 24 | 32 | 4.92 (0.73) |
| **Hb (×g/L)** | Baseline | 50 | 140.42 (21.62) |
|  | Month 3 | 47 | 134.23 (18.88) |
|  | Month 6 | 43 | 140.32 (20.31) |
|  | Month 12 | 38 | 142.16 (18.59) |
|  | Month 24 | 32 | 144.25 (21.99) |
| **PLT (×10^9^/L)** | Baseline | 50 | 197.84 (58.39) |
|  | Month 3 | 47 | 205.94 (50.53) |
|  | Month 6 | 43 | 197.60 (49.33) |
|  | Month 12 | 38 | 191.42 (51.68) |
|  | Month 24 | 32 | 187.61 (70.20) |
| **TBIL (μmol/L)** | Baseline | 50 | 18.05 (10.47) |
|  | Month 3 | 47 | 13.93 (5.78) |
|  | Month 6 | 43 | 16.58 (7.62) |
|  | Month 12 | 37 | 16.42 (8.79) |
|  | Month 24 | 32 | 17.08 (7.35) |
| **AST (U/L)** | Baseline | 50 | 29.54 (10.65) |
|  | Month 3 | 47 | 27.67 (9.75) |
|  | Month 6 | 41 | 31.57 (12.44) |
|  | Month 12 | 36 | 27.24 (9.93) |
|  | Month 24 | 32 | 26.88 (10.94) |
| **ALT (U/L)** | Baseline |  | 35.81 (23.57) |
|  | Month 3 | 47 | 26.01 (15.61) |
|  | Month 6 | 42 | 32.10 (27.19) |
|  | Month 12 | 37 | 29.01 (23.28) |
|  | Month 24 | 32 | 28.71 (23.58) |
| **LDH (U/L)** | Baseline |  | 225.08 (64.18) |
|  | Month 3 | 47 | 226.91 (47.08) |
|  | Month 6 | 38 | 219.58 (40.97) |
|  | Month 12 | 29 | 223.19 (36.42) |
|  | Month 24 | 24 | 214.33 (63.88) |
| **Scr (μmol/L)** | Baseline | 50 | 104.07 (48.22) |
|  | Month 3 | 47 | 91.74 (33.87) |
|  | Month 6 | 45 | 93.87 (36.92) |
|  | Month 12 | 36 | 90.90 (40.19) |
|  | Month 24 | 33 | 98.53 (40.10) |
| **INR** | Baseline | 50 | 1.11 (0.33) |
|  | Month 3 | 47 | 1.94 (0.35) |
|  | Month 6 | 46 | 2.00 (0.59) |
|  | Month 12 | 38 | 2.07 (0.54) |
|  | Month 24 | 34 | 2.06 (0.53) |

Abbreviations: ALT, alanine transaminase; AST, aspartate transaminase; Hb, haemoglobin; INR, international normalised ratio; LDH, lactate dehydrogenase; PLT, platelet; RBC, red blood cell; SCr, serum creatinine; SD, standard deviation; TBIL, total bilirubin.

**Table S3 Linear Mixed-Effected Regression Analyses of Quality of Life Change**

| **Measure** | **Parameter** | **Estimated** | **SE** | **95% CI** | **Sig.** |
| --- | --- | --- | --- | --- | --- |
| **KCCQ Overall Summary Score** | Baseline Score (Intercept) | 30.29 | 3.22 | (23.80, 36.78) | <0.001 |
|  | *Change from Baseline at:* | | | | |
|  | Month 3 | +46.67 | 4.04 | (38.63, 54.71) | <0.001 |
|  | Month 6 | +52.51 | 4.21 | (44.15, 60.88) | <0.001 |
|  | Month 12 | +55.98 | 3.99 | (48.04, 63.92) | <0.001 |
|  | Month 24 | +57.45 | 3.94 | (49.59, 65.31) | <0.001 |
| **EQ-5D-3L Total Score** | Baseline Score (Intercept) | 10.16 | 0.42 | (9.32, 11.00) | <0.001 |
|  | *Change from Baseline at:* | | | | |
|  | Month 3 | -3.84 | 0.47 | (-4.78, -2.90) | <0.001 |
|  | Month 6 | -3.96 | 0.48 | (-4.93, -3.00) | <0.001 |
|  | Month 12 | -4.48 | 0.45 | (-5.39, -3.58) | <0.001 |
|  | Month 24 | -4.70 | 0.44 | (-5.58, -3.82) | <0.001 |
| **EQ-5D VAS Score** | Baseline Score (Intercept) | 41.92 | 12.55 | (10.37, 73.47) | <0.001 |
|  | *Change from baseline at:* | | | | |
|  | Month 3 | +39.50 | 4.19 | (31.14, 47.87) | <0.001 |
|  | Month 6 | +38.10 | 4.02 | (30.05, 46.15) | <0.001 |
|  | Month 12 | +39.56 | 4.17 | (31.23, 47.88) | <0.001 |
|  | Month 24 | +40.19 | 4.08 | (32.02, 48.35) | <0.001 |

Abbreviations: CI, confidence interval; EQ-5D-3L, EuroQol 5-dimension 3-level questionnaire; EQ-5D VAS, EQ-5D visual analogue scale; KCCQ, Kansas City Cardiomyopathy Questionnaire; SE, standard error.

**Supplementary Figure**

**Figure S1 Corheart 6 Head Pressure Flow Rate (HQ) Curves.**

**Figure S2 Prospective, Multi-Centre Study Flow Diagram**

**Figure S3 The Outlet Graft sleeve of Corheart 6 Left Ventricular Assist System**

**Figure S4 Cumulative Incidence of Right Heart Failure**

**Figure S5 Cumulative Incidence of Driveline Infection**

**Figure S6 Dynamic changes in lactate dehydrogenase (LDH) levels from baseline to 24 months post-implantation.**

**Figure S7 Distribution of International Normalized Ratio (INR) values at baseline and at different follow-up visit (1-month, 3-month, 6-month, 12-month and 24-month) post-implantation.**
